# Supplementary material for: The Effect of Antibiotics Treatment on the Maternal Immune Response and Gut Microbiome in Pregnant and Non-Pregnant Mice
Source: Nutrients. 2023 Jun 12;15(12):2723. doi: 10.3390/nu15122723 (PMC10300872; doi:10.3390/nu15122723)
Supplement: Supplementary file 1 [file nutrients-15-02723-s001.zip › nutrients-2373220-supplementary.pdf]

Supplementary files:

Supplementary table S1: Antibodies used for staining Th cell subsets in the spleen, PP and MLN.

Marker	Fluorochrome	Dilution	Supplier	mix
CD4	PerCp-Cy5.5	75x	Biolegend	Extracellular
CD3	BV605	25x	Biolegend	Extracellular
Tbet	BV421	10x	Biolegend	Intracellular
RORgT	PE	100x	eBioscience	Intracellular
Gata3	AF647	100x	BD Pharmingen	Intracellular
FoxP3	Fitc	50x	eBioscience	intracellular
Dead/Live	Zombie NIR	1000x	Biolegend	

Supplementary table S2: Antibodies used for staining of dendritic cells.

Marker	Fluorochrome	Dilution	supplier
MHCII	PerCp-Cy5.5	1:200	Biolegend
CD11c	APC	1:50	BD Pharmingen
CD64	PE-Cy7	1:25	Biolegend
CD19	FITC	1:25	Biolegend
B220	FITC	1:25	Biolegend
Nkp46	FITC	1:25	Biolegend
CD103	BV421	1:20	Biolegend
CD11b	PE	1:50	Biolegend
Dead/live	Zombie green	1:1000	Biolegend

Supplementary table S3: Antibodies used for monocyte staining.

Marker	Fluorochrome	Dilution	Supplier
MHCII	PerCp-Cy5.5	200x	Biolegend
Ly6C	AF488	200x	Biolegend
CD43	APC	100x	Biolegend
CD11b	PE	50x	Biolegend
CD80	PB	25x	Biolegend
Ly6G	BV605	25x	BD Horizon

Figure S1A-C show the gating strategies for Th cell subsets, dendritic cell subsets and monocytes subsets:

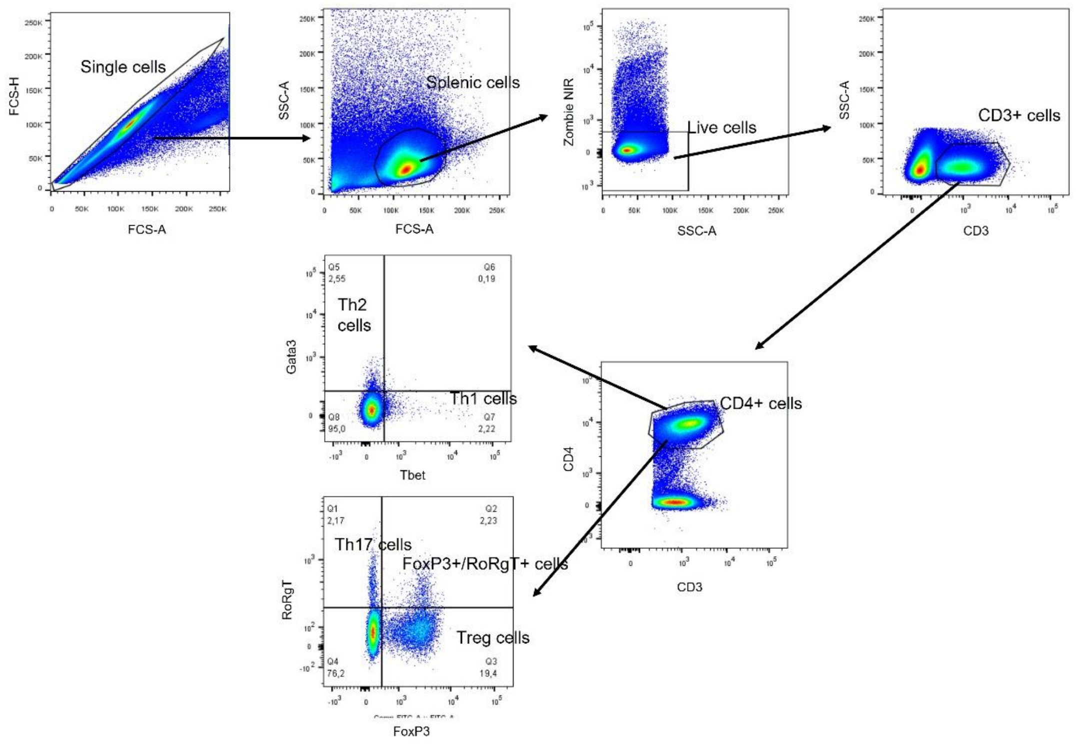

Figure S1A: Gating strategy of Th cell subsets: An example is shown for Th cell subsets in the Peyer's patches (PP). After selecting single cells, cells of the PP were gated based on size and scatter in the forward/side scatter plot. After selecting live cells, T cells were gated by selecting CD3$^+$ cells. Within the CD3$^+$ cells, CD4$^+$ were identified and within CD4$^+$ and population, percentages of cells expressing, Tbet (Th1 cells), GATA3 (Th2 cells), FoxP3 (Treg cells), RoRgT (Th17 cells) as well as FoxP3+/RoRgT+ double positive cells were assessed. Gates were set using FMOs.

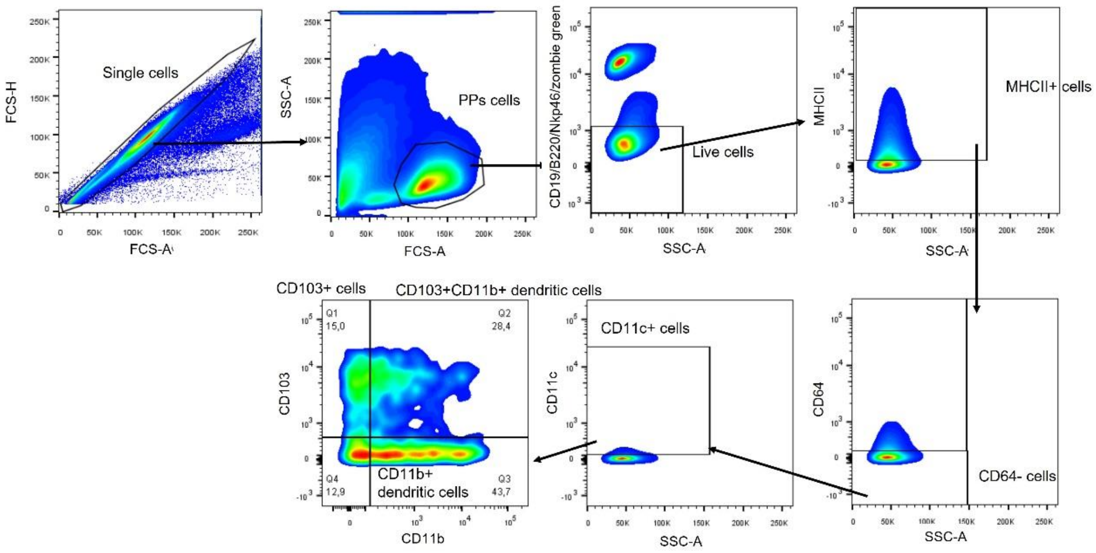

Figure S1B: Gating strategy of dendritic cells: An example is shown for gating of dendritic cells in the PP. To gate DCs, single cells were gated and cells of the PP were gated based on size and scatter in the forward/side scatter plot. After gating live cells, $MHCII^{+}$ cells were gated. Within these MHCII+ cells, CD64+ cells were macrophages. Within the MHCII+/CD64- gate, CD11c+ dendritic cells were selected. Within this CD11c+ population, we identified CD103+/CD11b- and CD103-/CD11b+ and CD103+/CD11b+ DC. Gates were set using FMOs.

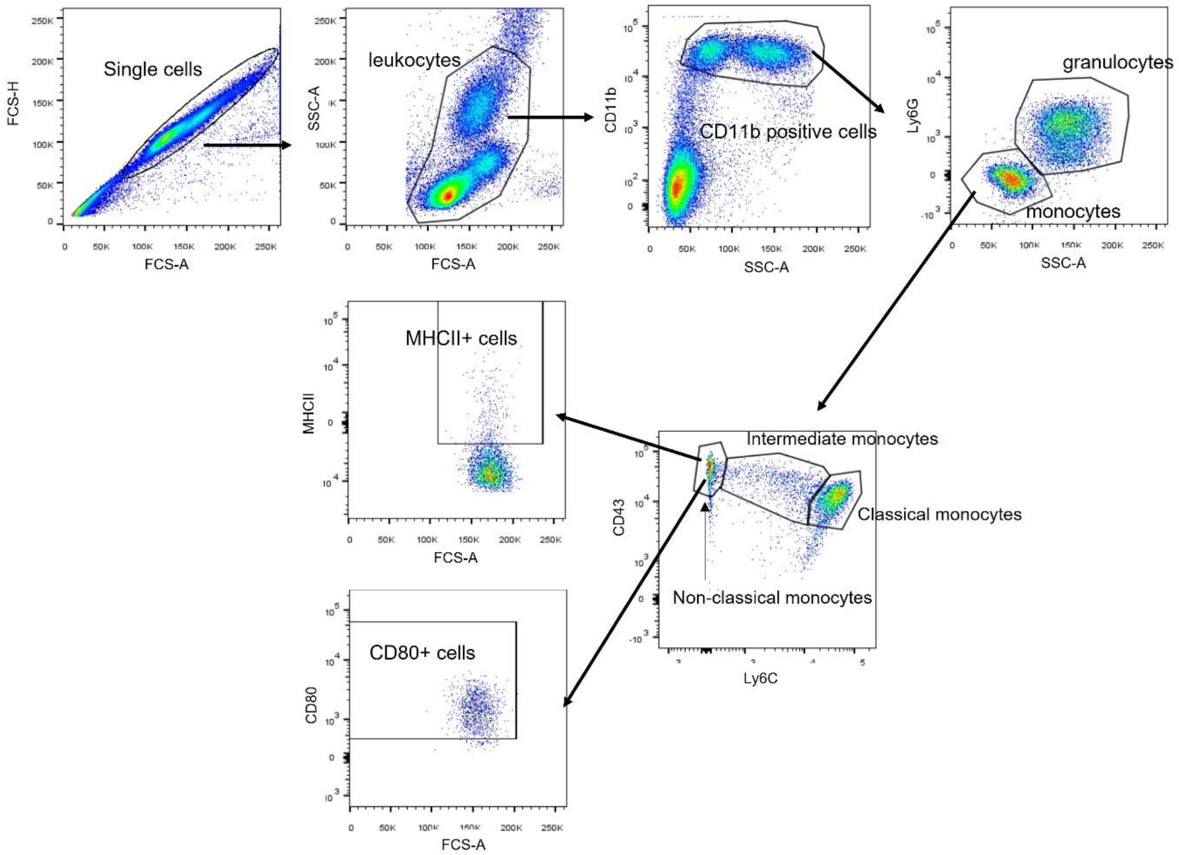

Figure S1C: Gating strategy of monocytes: After selecting single cells, blood leukocytes were first gated based on forward/side scatter characteristics. Then the CD11b positive cell population was selected and monocytes were selected by gating on Ly6G negative cells. Classical, intermediate and non-classical monocytes were selected based on their CD43 and LY6C expression. In each monocyte subpopulation, we evaluated expression of MHCII and CD80 using FMOs to set the gates (figure shows example for selection of MHCII+ and CD80+ non-classical monocytes).

Figure S2:

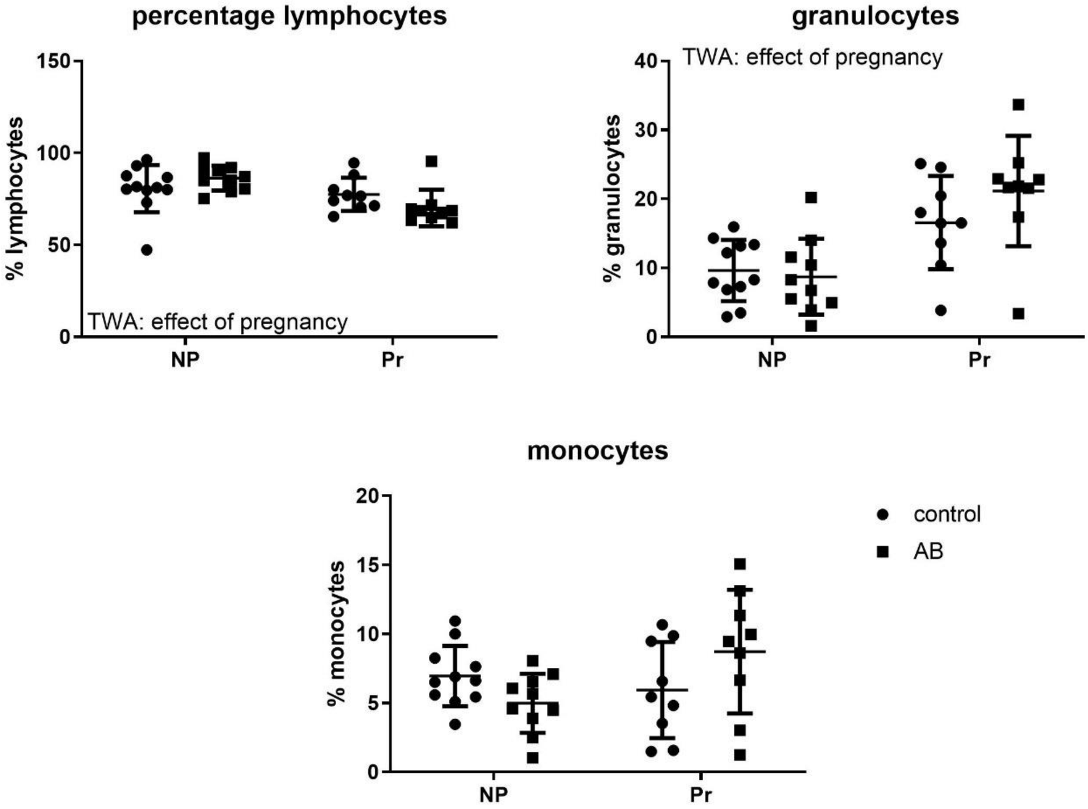

Figures S2: Peripheral blood leukocyte subpopulations in control and antibiotics treated pregnant and non-pregnant mice.
Pregnant AB treated mice: n=9; untreated pregnant mice: n=9; non-pregnant AB treated mice: n=10; untreated non-pregnant mice: n=11.
*:Two-way ANOVA was done for all subsets followed by Sidak's multiple comparison's test, $p<0.05$.

The percentages of lymphocytes and granulocytes within the total leukocyte population were affected by pregnancy (TWA, $p<0.05$). Lymphocytes were decreased, while granulocyte were increased during pregnancy. No effect of antibiotics was found on the leukocyte subpopulations in pregnant or non-pregnant mice (TWA, $p<0.05$).

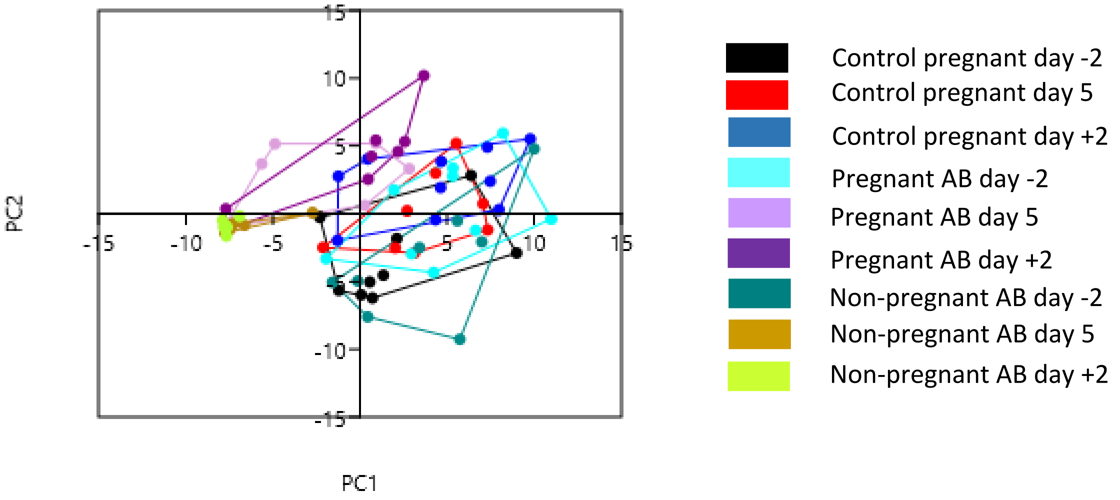

Figure S3: PCA plot of bacterial genera in control pregnant mice, pregnant AB treated mice and non-pregnant AB-treated mice before, during and after AB treatment. Pregnant AB treated mice: n=9; untreated pregnant mice: n=9; non-pregnant AB treated mice: n=8;

Table S4: The microbial genera explaining the differences in fecal microbiome in antibiotics treated pregnant mice compared with before the start of the antibiotics treatment.

Taxon	% contribution to variation due to AB treatment	Mean day 7 AB	Mean day 14 AB	Mean day 18 AB	Friedman followed by Dunn's test (day -2 vs day 5)	Friedman followed by Dunn's test (day -2 vs day +2)
Alistipes	26.97	3.39	45.4	20.9	ns	ns
Barnesiella	38.26	25.2	6.58	10.7	*	*
Faecalibaculum	49.26	11.2	14.6	13.9	ns	ns
Lachnoclostridium	55.47	9.7	5.14	5.87	*	ns
Escherichia	61.48	0.25	0.89	10.9	ns	ns
Bacteroides	64.55	1.99	1.17	5.08	*	ns
Lactobacillus	66.77	1.27	2.7	1.65	ns	ns
Akkermansia	68.97	2.4	2.46	0.774	ns	ns
Flintibacter	71.06	2.62	0.941	3.17	ns	ns
Acetatifactor	72.93	2.88	1.82	1.87	*	ns
Parasutterella	74.79	3.49	0.86	0.992	ns	ns
unclassified Bacteroidales	76.58	3.72	0.797	0.826	*	*
Mucispirillum	79.78	2.31	0.681	1.43	ns	ns
Intestinimonas	81.09	1.46	0.654	2.23	ns	ns
Alloprevotella	82.21	2.28	0.406	0.337	*	*
Oscillibacter	83.28	1.3	0.588	1.65	ns	ns
Clostridium	84.27	1.62	0.89	1.55	ns	ns
Christensenella	85.02	1.2	0.37	0.573	ns	ns
Bifidobacterium	85.74	0.267	0.598	1.24	ns	ns
Ruminiclostridium	86.4	0.867	0.517	1.21	ns	ns
Odoribacter	86.97	1.06	0.361	0.32	ns	*
Staphylococcus	87.55	0.00305	1.08	0.0498	ns	ns
Allobaculum	88.08	0.434	0.717	0.686	ns	ns
Romboutsia	88.6	0.24	0.587	0.557	ns	ns
Tyzzerella	89.11	0.901	0.411	0.52	ns	ns
Oscillospira	89.61	0.584	0.248	0.795	ns	ns
Sphingobium	90.08	0.212	0.743	0.391	ns	ns

Desulfovibrio	90.53	0.637	0.523	0.435	ns	ns
unclassified Clostridiales	90.98	0.924	0.139	0.271	ns	ns
Bacillus	91.39	0.00739	0.705	0.108	ns	ns
Anaerocolumna	91.78	0.748	0.124	0.143	*	ns
Catabacter	92.17	0.472	0.27	0.374	ns	ns
Salmonella	92.5	0.0077	0.559	0.0798	ns	ns
Vallitalea	92.82	0.422	0.314	0.51	ns	ns
Coprobacter	93.14	0.548	0.16	0.199	*	ns
Anaerotaenia	93.45	0.411	0.162	0.4	ns	ns
Turicibacter	93.76	0.291	0.218	0.394	ns	ns
Anaerotruncus	94.02	0.409	0.18	0.302	ns	ns
Anaeroplasma	94.27	0.462	0.13	0.165	ns	ns
Acetivibrio	94.52	0.437	0.149	0.359	*	ns
Dorea	94.77	0.23	0.135	0.445	ns	ns
Candidatus Soleaferrea	95.01	0.386	0.147	0.293	ns	ns
Roseburia	95.24	0.223	0.184	0.43	ns	ns
unclassified Cyanobacteria	95.47	0.354	0.0841	0.152	ns	ns
Saccharofermentans	95.67	0.349	0.093	0.218	*	ns
Bilophila	95.85	0.216	0.0822	0.237	ns	ns
Hydrogenoanaerobacterium	96.02	0.193	0.113	0.293	ns	ns
Ruminococcus	96.18	0.245	0.141	0.206	ns	ns
Robinsoniella	96.34	0.303	0.066	0.0695	ns	ns
Rhodospirillum	96.48	0.21	0.0801	0.129	ns	ns
Rikenella	96.62	0.266	0.0397	0.0518	*	ns
Enterococcus	96.75	0.0185	0.198	0.0572	ns	ns
Pseudomonas	96.87	0.00706	0.194	0.0677	ns	ns
Prevotella	96.98	0.224	0.0136	0.0135	*	*
Marvinbryantia	97.09	0.206	0.0466	0.0758	ns	ns
Dehalobacterium	97.2	0.148	0.0697	0.177	ns	ns
unclassified Burkholderiales	97.31	0.212	0.0135	0.00627	*	ns
Tannerella	97.42	0.216	0.0146	0.017	*	*
Coprococcus	97.52	0.1	0.104	0.14	ns	ns
Blautia	97.62	0.167	0.0886	0.159	ns	ns
Butyrivibrio	97.72	0.079	0.0587	0.154	ns	ns
Pseudoflavonifractor	97.82	0.152	0.0617	0.11	ns	ns
Gracilibacter	97.91	0.147	0.0739	0.116	*	ns
Stomatobaculum	98	0.0885	0.0491	0.124	ns	ns
Ruthenibacterium	98.08	0.0501	0.0275	0.137	ns	ns
Anaeromassilibacillus	98.16	0.0938	0.0812	0.0983	ns	ns
Anaerobacterium	98.23	0.0988	0.0911	0.128	ns	ns
unclassified Clostridiales Family XIII. Incertae Sedis	98.31	0.0937	0.0462	0.1	ns	ns
unclassified Lachnospiraceae	98.38	0.143	0.0433	0.0508	*	ns

Eisenbergiella	98.44	0.13	0.0325	0.0677	ns	ns
Syntrophococcus	98.51	0.0668	0.0664	0.0265	ns	ns
unclassified Erysipelotrichales	98.57	0.1	0.0585	0.0342	ns	*
unclassified Clostridia	98.64	0.0642	0.0209	0.0942	*	ns
Anaerostipes	98.69	0.115	0.0194	0.0131	*	*
Flavonifractor	98.74	0.0959	0.0139	0.0233	*	ns
Natranaerovirga	98.79	0.0946	0.0309	0.0456	ns	ns
Enterorhabdus	98.84	0.0112	0.0758	0.0392	ns	ns
Defluviitalea	98.89	0.0744	0.0217	0.0272	ns	ns
Hungatella	98.93	0.0715	0.0189	0.0528	ns	ns
Fournierella	98.98	0.0527	0.0187	0.0599	ns	ns
unclassified Betaproteobacteria	99.01	0.0701	0.0127	0.00507	ns	ns
Murimonas	99.05	0.0474	0.0358	0.0497	ns	ns
Parvibacter	99.09	0.0109	0.0543	0.0336	ns	ns
Cryptanaerobacter	99.12	0.0664	0.0135	0.026	ns	ns
Oribacterium	99.15	0.0582	0.00823	0.00412	*	*
Alkalibaculum	99.18	0.0244	0.0202	0.0359	ns	ns
Alkalibacter	99.2	0.0491	0.00746	0.01	ns	ns
Butyricicoccus	99.23	0.0383	0.0119	0.0265	ns	ns
Anaerovorax	99.26	0.0322	0.0146	0.0243	ns	ns
unclassified Erysipelotrichia	99.28	0.0444	0.01	0.0157	ns	ns
Mobilitalea	99.3	0.0432	0.00957	0.0047	ns	*
Herbinix	99.32	0.0445	0.00496	0.00432	*	*
Massilia	99.35	0.0397	0.00843	0.00305	ns	ns
Mageeibacillus	99.37	0.0149	0.0113	0.0355	ns	ns
unclassified Eubacteriaceae	99.39	0.0332	0.00781	0.0231	ns	ns
Cohaesibacter	99.41	0.00902	0.0318	0.00912	ns	ns
Filifactor	99.43	0.0295	0.00849	0.0172	ns	ns
Alkaliphilus	99.45	0.0328	0.00719	0.0162	ns	ns
Peptococcus	99.47	0.0291	0.0133	0.0161	ns	ns
Herbaspirillum	99.49	0.0356	0.00298	0.00169	ns	ns
Sporobacter	99.51	0.0366	0.00849	0.0138	*	ns
unclassified Porphyromonadaceae	99.53	0.0152	0.00746	0.0191	ns	ns
Falcatimonas	99.54	0.0249	0.0127	0.0144	ns	ns
Kopriimonas	99.56	0.027	0.00482	0.00814	ns	ns
unclassified Clostridiaceae	99.58	0.0313	0.00272	0.00186	ns	ns
Olsenella	99.59	0.0239	0.0142	0.00408	ns	ns
Abyssivirga	99.61	0.0156	0.00392	0.0201	ns	ns
Falsiporphyromonas	99.62	0.0241	0.00282	0.00466	ns	ns
Sutterella	99.63	0.0185	0.00531	0.00792	ns	ns
Nocardioides	99.65	0.0254	0	0	ns	ns

Fusicatenibacter	99.66	0.0225	0.00423	0.00349	ns	ns
Intestinibacter	99.67	0.0191	0	0.00711	ns	ns
Faecalibacterium	99.68	0.0178	0.00444	0.0121	ns	ns
Hespellia	99.69	0.0183	0.00648	0.00729	ns	ns
unclassified Candidatus Saccharibacteria	99.7	0.0163	0.00896	0.00487	ns	ns
Paeniclostridium	99.72	0.0171	0.000941	0.0061	ns	ns
unclassified Alphaproteobacteria	99.73	0.02	0.00369	0.00819	*	ns
Papillibacter	99.74	0.0186	0.00583	0.00643	ns	ns
unclassified Ruminococcaceae	99.75	0.0164	0.00561	0.00102	ns	ns
Anaerosporobacter	99.76	0.0125	0.00765	0.01	ns	ns
Anaerobium	99.76	0.0142	0.00402	0.00351	ns	ns
Chloroflexus	99.77	0.0158	0.00207	0.00161	ns	ns
unclassified Paenibacillaceae	99.78	0.0148	0.00207	0.00242	ns	ns
Acetanaerobacterium	99.79	0.0119	0.00934	0.00504	ns	ns
Corynebacterium	99.8	0.00107	0.00181	0.0149	ns	ns
Desulfonatronum	99.81	0.0131	0.00198	0.0027	ns	ns
Azoarcus	99.81	0.015	0	0	ns	ns
Erysipelatoclostridium	99.82	0.00386	0.00456	0.00999	ns	ns
Lachnoanaerobaculum	99.83	0.0134	0.00104	0.000746	ns	ns
Chitinimonas	99.83	0.0131	0.00104	0	ns	ns
Holdemania	99.84	0.00918	0.000909	0.00574	ns	ns
unclassified Erysipelotrichaceae	99.85	0.005	0.00276	0.00724	ns	ns
Kluyvera	99.85	0.00493	0	0.00738	ns	ns
Ethanoligenens	99.86	0.01	0.00336	0	ns	ns
Candidatus Stoquefichus	99.86	0.00388	0.00285	0.00661	ns	ns
Dysgonomonas	99.87	0.0099	0.00185	0.00102	ns	ns
Caproiciproducens	99.87	0.00948	0.000941	0.00102	ns	ns
Asaccharospora	99.88	0.00773	0	0.00305	ns	ns
Propionivibrio	99.88	0.00931	0.00104	0	ns	ns
unclassified Anaeroplasmatales	99.89	0.00685	0	0.00386	ns	ns
unclassified Mollicutes	99.89	0.00412	0.00444	0.00444	ns	ns
Breznakia	99.9	0.00382	0.00262	0.00623	ns	ns
Methyloversatilis	99.9	0.00836	0	0.000807	ns	ns
Azospira	99.91	0.008	0	0.00162	ns	ns

Casaltella	99.91	0.00107	0	0.00752	ns	ns
Desulfitobacterium	99.92	0.00574	0.00182	0.00405	ns	ns
Aestuariispira	99.92	0.00491	0.00275	0.00284	ns	ns
Acetobacter	99.92	0.00708	0	0	ns	ns
Methylophilus	99.93	0.00708	0	0	ns	ns
unclassified Peptostreptococcaceae	99.93	0.00384	0.00104	0.00305	ns	ns
Lutispora	99.93	0.00595	0.000941	0	ns	ns
Parapedobacter	99.94	0.00587	0	0	ns	ns
Terrisporobacter	99.94	0.00383	0	0.00203	ns	ns
Herbivorax	99.94	0.00291	0.00181	0.00183	ns	ns
Paraeggerthella	99.95	0	0.00419	0.00186	ns	ns
Natronincola	99.95	0.00377	0.00104	0.00102	ns	ns
Thermotalea	99.95	0.0039	0.00195	0	ns	ns
unclassified Deltaproteobacteria	99.95	0.00297	0.00198	0.000807	ns	ns
Thauera	99.96	0.00396	0	0.000841	ns	ns
Clostridioides	99.96	0.00391	0.000941	0	ns	ns
Sporanaerobacter	99.96	0.00288	0.00195	0	ns	ns
Variovorax	99.96	0.00412	0	0	ns	ns
Catonella	99.96	0.00315	0	0.00102	ns	ns
Serpentinicella	99.97	0.00192	0.00207	0	ns	ns
Schlegelella	99.97	0.00315	0	0.000841	ns	ns
Acetoanaerobium	99.97	0.00295	0	0.00102	ns	ns
Kiloniella	99.97	0.000971	0.00194	0.00102	ns	ns
Rubrivivax	99.97	0.00203	0.000909	0.000873	ns	ns
Parasporobacterium	99.97	0.00186	0	0.00171	ns	ns
Caldimonas	99.98	0.0031	0	0	ns	ns
Pedobacter	99.98	0.00196	0.00147	0	ns	ns
Candidatus Vestibaculum	99.98	0.00289	0	0	ns	ns
Caloranaerobacter	99.98	0.00288	0	0	ns	ns
Brassicibacter	99.98	0.00288	0	0	ns	ns

Sphingobacterium	99.98	0.000971	0.00104	0.00102	ns	ns
Lachnospira	99.98	0.000991	0	0.00183	ns	ns
unclassified Bacteroidia	99.99	0.00191	0.000909	0	ns	ns
Gemmiger	99.99	0.00191	0.000732	0	ns	ns
Mycobacterium	99.99	0.000961	0	0.00149	ns	ns
Atopobium	99.99	0.00208	0	0	ns	ns
Desulfotomaculum	99.99	0.00206	0	0	ns	ns
Vibrio	99.99	0.00194	0	0	ns	ns
Comamonas	99.99	0.00194	0	0	ns	ns
Mangroviflexus	99.99	0.00188	0	0	ns	ns
Proteiniborus	99.99	0.000991	0.000941	0	ns	ns
Insolitispirillum	100	0	0	0.00182	ns	ns
Fusibacter	100	0.000971	0.000941	0	ns	ns
Aerococcus	100	0	0.00104	0	ns	ns
Shuttleworthia	100	0.000991	0	0	ns	ns
Moryella	100	0.000991	0	0	ns	ns
Fodinicurvata	100	0.000971	0	0	ns	ns
unclassified Alcaligenaceae	100	0.000971	0	0	ns	ns
Oxobacter	100	0	0.000941	0	ns	ns
Geosporobacter	100	0.000916	0	0	ns	ns

*: Friedman followed by Dunn's multiple comparison test, $p<0.05$. ns: non-significant.

Table S5: The microbial genera explaining the differences in fecal microbiome in antibiotics treated non-pregnant mice compared with before the start of the antibiotics treatment.

Taxon	% contribution to variation due to AB treatment	Cumulative %	Mean day -2	Mean day 5	Mean day +2	Friedman followed by Dunn's test (day -2 vs day 5)	Friedman followed by Dunn's test (day -2 vs day +2)
Alistipes	34.07	34.07	2.27	59	62	*	*
Bacteroides	18.24	52.31	2.5	20.7	30.8	ns	ns
Barnesiella	13.78	66.1	31.6	2.58	0.152	*	*
Faecalibaculum	7.513	73.61	10.2	9.8	0.208	ns	ns
Akkermansia	3.898	77.51	4.94	1.34	4.1	ns	ns
Lachnoclostridium	2.969	80.48	6.67	0.761	0.114	*	*
Parasutterella	2.423	82.9	5.49	0.396	0.00601	*	*
Acetatifactor	1.407	85.93	3.18	0.271	0.0679	ns	ns
unclassified Bacteroidales	1.327	87.25	3.04	0.157	0.0282	*	*
Allobaculum	1.097	88.35	2.15	0.648	0.0504	ns	ns
Alloprevotella	0.9548	89.31	2.17	0.09	0.00691	*	*
Flintibacter	0.8618	90.17	1.97	0.263	0.144	*	ns
Oscillibacter	0.7039	90.87	1.56	0.213	0.061	ns	ns
Escherichia	0.604	91.48	0.00219	0.0485	1.41	ns	ns
Clostridium	0.5441	92.02	1.25	0.209	0.0438	*	ns
Intestinimonas	0.5171	92.54	1.16	0.185	0.0954	ns	ns
Mucispirillum	0.4666	93	1.04	0.128	0.011	ns	ns
Ruminiclostridium	0.3296	93.33	0.758	0.175	0.0297	ns	ns
Tyzzerella	0.3158	93.65	0.727	0.0609	0.019	*	*
Bifidobacterium	0.2894	93.94	0.393	0.417	0.056	ns	ns
Sphingobium	0.2682	94.21	0.291	0.376	0.00748	ns	ns
Desulfovibrio	0.2592	94.47	0.568	0.12	0.0154	ns	*
Vallitalea	0.2548	94.72	0.586	0.0633	0.0101	ns	ns
Oscillospira	0.2487	94.97	0.558	0.0815	0.0237	ns	ns
Coprobacter	0.2412	95.21	0.548	0.0459	0.00161	*	*
unclassified Clostridiales	0.2179	95.43	0.503	0.0415	0.015	*	ns
Lactobacillus	0.1918	95.62	0.348	0.216	0.0208	ns	ns
Anaerotruncus	0.1901	95.81	0.423	0.0554	0.0107	ns	ns
unclassified Erysipelotrichales	0.1751	95.99	0.396	0.0368	0.0034	*	*
Acetivibrio	0.1662	96.15	0.384	0.0474	0.00599	*	ns
Dorea	0.1657	96.32	0.372	0.0518	0.00991	ns	ns
unclassified Burkholderiales	0.1502	96.47	0.341	0.00739	0	*	*
Ruminococcus	0.1462	96.61	0.337	0.0342	0.0123	*	ns
Odoribacter	0.14	96.75	0.311	0.0435	0.00577	ns	ns
Prevotella	0.1225	96.88	0.278	0.00569	0.00354	ns	*
unclassified Cyanobacteria	0.1215	97	0.267	0.0277	0.00256	ns	ns

Anaerotaenia	0.1026	97.1	0.233	0.0361	0.00169	*	ns
Rhodospirillum	0.1017	97.2	0.211	0.0452	0	ns	*
Tannerella	0.0979	97.3	0.223	0.0122	0	*	*
Roseburia	0.09645	97.4	0.207	0.0625	0.00758	ns	ns
Candidatus Soleaferrea	0.09182	97.49	0.204	0.0502	0.0156	ns	ns
Turicibacter	0.09037	97.58	0.179	0.069	0.00512	ns	*
Rikenella	0.09027	97.67	0.207	0.00648	0.00334	*	*
unclassified Lachnospiraceae	0.09011	97.76	0.205	0.016	0.00514	*	*
Hydrogenoanaerobacterium	0.08678	97.85	0.195	0.0287	0.00495	ns	ns
Robinsoniella	0.08374	97.93	0.191	0.0105	0.00242	*	*
Anaerocolumna	0.08001	98.01	0.178	0.0239	0.00728	ns	*
Blautia	0.07855	98.09	0.183	0.0203	0.011	*	ns
Anaeroplasma	0.07422	98.16	0.166	0.00701	0.00684	ns	ns
unclassified Betaproteobacteria	0.07238	98.23	0.164	0.00917	0.000811	*	*
Christensenella	0.07214	98.31	0.172	0.0354	0.0142	ns	ns
Saccharofermentans	0.06642	98.37	0.15	0.00553	0.00915	ns	ns
Marvinbryantia	0.06441	98.44	0.145	0.0151	0.00243	ns	*
Eisenbergiella	0.06224	98.5	0.139	0.011	0	ns	*
Coprococcus	0.06146	98.56	0.139	0.0143	0.00334	ns	ns
Dehalobacterium	0.05634	98.62	0.119	0.0343	0.00326	ns	ns
Stomatobaculum	0.0544	98.67	0.12	0.0157	0.0122	*	ns
Mobilitalea	0.05345	98.73	0.122	0.00262	0	*	*
Romboutsia	0.05153	98.78	0.0955	0.0337	0.0131	ns	ns
Anaerobacterium	0.05044	98.83	0.106	0.0307	0	ns	ns
Bilophila	0.04971	98.88	0.112	0.0161	0.00413	ns	ns
Anaeromassilibacillus	0.04782	98.93	0.106	0.0127	0.000863	ns	ns
unclassified Clostridiales Family XIII. Incertae Sedis	0.04687	98.97	0.107	0.0107	0.00167	ns	ns
Anaerostipes	0.04461	99.02	0.102	0.00424	0.00261	*	*
Pseudoflavonifractor	0.04378	99.06	0.0996	0.00995	0.00417	*	ns
Gracilibacter	0.04368	99.1	0.0982	0.0163	0.00411	ns	ns
Oribacterium	0.03465	99.14	0.0787	0.00412	0	*	*
Natranaerovirga	0.03204	99.17	0.0722	0.00628	0	*	ns
Flavonifractor	0.03173	99.2	0.0721	0.000875	0.00167	*	*
Massilia	0.02901	99.23	0.0659	0	0	*	*
Cohaesibacter	0.02854	99.26	0.0166	0.056	0	ns	ns
unclassified Erysipelotrichia	0.02706	99.29	0.0611	0.00262	0.00163	ns	*
Murimonas	0.02618	99.31	0.0566	0.0101	0.00334	ns	ns
Hungatella	0.02577	99.34	0.0567	0.00552	0.00331	ns	ns
Cryptanaerobacter	0.0256	99.36	0.057	0.00525	0.000816	ns	ns
Catabacter	0.025	99.39	0.0431	0.0233	0.0203	ns	ns
unclassified Eubacteriaceae	0.0212	99.41	0.0481	0.00163	0	*	*
Nocardioides	0.02057	99.43	0.0468	0	0	*	*
unclassified Clostridiaceae	0.02035	99.45	0.0462	0.00156	0	*	*

Peptococcus	0.01982	99.47	0.0436	0.00484	0.000807	ns	ns
Herbinix	0.0198	99.49	0.0446	0.00175	0	*	*
Olsenella	0.01908	99.51	0.0375	0.00983	0	ns	ns
Fusicatenibacter	0.01631	99.53	0.0371	0.000722	0	*	*
Enterorhabdus	0.01582	99.54	0.0111	0.0295	0	ns	ns
Hespellia	0.01572	99.56	0.0359	0.00238	0	*	*
Sutterella	0.01558	99.57	0.0355	0.000875	0.000816	*	*
Butyricicoccus	0.01462	99.59	0.0318	0.00324	0	ns	ns
Falsiporphyromonas	0.01433	99.6	0.0322	0	0.000807	ns	ns
Ruthenibacterium	0.01372	99.62	0.0266	0.00665	0.00565	ns	ns
Sporobacter	0.01267	99.63	0.0287	0.000875	0	*	*
Butyrivibrio	0.01237	99.64	0.019	0.0144	0.00332	ns	ns
Anaerovorax	0.0121	99.65	0.0252	0.00507	0.000861	ns	ns
Abyssivirga	0.01192	99.67	0.0276	0.00315	0.000807	*	ns
unclassified Ruminococcaceae	0.0116	99.68	0.0264	0	0	*	*
Chitinimonas	0.01115	99.69	0.0243	0.00175	0	ns	ns
unclassified Clostridia	0.01088	99.7	0.0212	0.00237	0.00651	*	ns
Falcatimonas	0.0105	99.71	0.0235	0.00405	0	ns	ns
Fournierella	0.01033	99.72	0.0127	0.013	0	ns	ns
Alkalibaculum	0.009789	99.73	0.0205	0.00397	0	ns	ns
Azoarcus	0.009532	99.74	0.0217	0	0	ns	ns
Herbaspirillum	0.008934	99.75	0.0201	0.000875	0	*	*
unclassified Porphyromonadaceae	0.008873	99.76	0.0192	0.00342	0	ns	ns
unclassified Candidatus Saccharibacteria	0.008775	99.77	0.0171	0.00623	0	ns	ns
Papillibacter	0.008263	99.77	0.0178	0.00342	0	*	*
unclassified Alphaproteobacteria	0.008213	99.78	0.0168	0.00775	0.00604	ns	ns
Erysipelatoclostridium	0.008022	99.79	0.0134	0.00818	0	ns	ns
Kopriimonas	0.007921	99.8	0.0177	0.00167	0	*	*
unclassified Paenibacillaceae	0.007059	99.81	0.0153	0.00167	0	ns	ns
Intestinibacter	0.006989	99.81	0.0155	0.000875	0	ns	ns
Desulfonatronum	0.006851	99.82	0.0152	0.0008	0	ns	ns
Azospira	0.006673	99.83	0.0135	0.0041	0	*	*
Parvibacter	0.006554	99.83	0.00861	0.00826	0.00244	ns	ns
Defluviitalea	0.006358	99.84	0.0142	0.00318	0.00167	*	ns
Acetanaerobacterium	0.006201	99.85	0.0122	0.00162	0.00248	ns	ns
Enterococcus	0.005895	99.85	0.00938	0.0061	0.00163	ns	ns
Faecalibacterium	0.005825	99.86	0.00923	0.00408	0.00757	ns	ns
unclassified Erysipelotrichaceae	0.005421	99.86	0.0117	0.00149	0	ns	ns
Syntrophococcus	0.00524	99.87	0.0114	0.000747	0	ns	ns
Lachnoanaerobaculum	0.005014	99.87	0.0114	0	0	ns	ns
Candidatus Vestibaculum	0.004872	99.88	0.0111	0	0	ns	ns
Kluyvera	0.004766	99.88	0	0	0.0115	ns	ns

Anaerosporobacter	0.00459	99.89	0.00951	0.00175	0	ns	ns
Methyloversatilis	0.004391	99.89	0.00997	0	0	ns	ns
Atopobium	0.00433	99.9	0.00984	0	0	ns	ns
Alkalibacter	0.00431	99.9	0.0061	0.00502	0	ns	ns
unclassified Mollicutes	0.004144	99.9	0.00942	0	0	ns	ns
Ethanoligenens	0.003927	99.91	0.00892	0	0	ns	ns
Lutispora	0.003814	99.91	0.00867	0	0	ns	ns
Alkaliphilus	0.003804	99.92	0.00533	0.00422	0.000807	ns	ns
Mageeibacillus	0.003792	99.92	0.00602	0.00227	0.00242	ns	ns
Rubrivivax	0.003588	99.92	0.00815	0	0	ns	ns
Holdemania	0.003581	99.93	0.00766	0.00162	0	ns	ns
Staphylococcus	0.003559	99.93	0.000952	0.00531	0.00439	ns	ns
Sphingobacterium	0.003408	99.93	0.00775	0	0	ns	ns
Desulfitobacterium	0.003282	99.94	0.00746	0	0	ns	ns
Acetobacter	0.002689	99.94	0.00611	0	0	ns	ns
Methylophilus	0.002576	99.94	0.00585	0	0	ns	ns
Parapedobacter	0.002543	99.94	0.00578	0	0	ns	ns
Insolitispirillum	0.002376	99.95	0.00435	0.00162	0	ns	ns
Candidatus Stoquefichus	0.002375	99.95	0.00504	0.000875	0	ns	ns
Aestuariispira	0.002337	99.95	0	0.00531	0	ns	ns
Caproiciproducens	0.002064	99.95	0.00469	0	0	ns	ns
Chloroflexus	0.002035	99.96	0.00203	0.0016	0.00161	ns	ns
Asaccharospora	0.001962	99.96	0.00389	0.000875	0	ns	ns
Hathewaya	0.001961	99.96	0.00399	0.000725	0	ns	ns
Paeniclostridium	0.001956	99.96	0.00388	0.000875	0	ns	ns
Casaltella	0.001914	99.96	0.00181	0.000725	0.00248	ns	ns
Dysgonomonas	0.001912	99.97	0.00435	0	0	ns	ns
unclassified Peptostreptococcaceae	0.001801	99.97	0.00409	0	0	ns	ns
Moryella	0.001752	99.97	0.00398	0	0	ns	ns
unclassified Alcaligenaceae	0.001743	99.97	0.00396	0	0	ns	ns
unclassified Anaeroplasmatales	0.001709	99.97	0.00389	0	0	ns	ns
Anaerobium	0.001664	99.97	0.00311	0.000875	0	ns	ns
Aerococcus	0.001656	99.98	0	0.0025	0.00162	ns	ns
Salmonella	0.001569	99.98	0.00121	0	0.00265	ns	ns
unclassified Bacteroidia	0.001555	99.98	0.00287	0.000752	0	ns	ns
Aminicella	0.00151	99.98	0.00276	0.000875	0	ns	ns
Caloranaerobacter	0.001411	99.98	0.00321	0	0	ns	ns
Brassicibacter	0.001411	99.98	0.00321	0	0	ns	ns
Kiloniella	0.001342	99.98	0.00305	0	0	ns	ns
Sideroxydans	0.001296	99.99	0.00294	0	0	ns	ns
Bacillus	0.001246	99.99	0.00283	0	0	ns	ns
Mycobacterium	0.00112	99.99	0.00193	0.0008	0	ns	ns
Comamonas	0.001064	99.99	0.00242	0	0	ns	ns
Herbivorax	0.000999	99.99	0.000905	0.00155	0	ns	ns

Caldimonas	0.0009638	99.99	0.00219	0	0	ns	ns
Gloeobacter	0.0009608	99.99	0.00218	0	0	ns	ns
Parasporobacterium	0.0009506	99.99	0.00216	0	0	ns	ns
Variovorax	0.000861	99.99	0.00196	0	0	ns	ns
Natronincola	0.0008588	99.99	0.00195	0	0	ns	ns
Shuttleworthia	0.0008193	100	0.00186	0	0	ns	ns
Terrisporobacter	0.0007469	100	0.000976	0	0.000863	ns	ns
Corynebacterium	0.0006915	100	0	0.0008	0.000919	ns	ns
Thermotalea	0.0005318	100	0.00121	0	0	ns	ns
Geosporobacter	0.000432	100	0.000981	0	0	ns	ns
Breznakia	0.0004294	100	0.000976	0	0	ns	ns
Schlegelella	0.000429	100	0.000975	0	0	ns	ns
Mangroviflexus	0.0004209	100	0.000957	0	0	ns	ns
Gemmiger	0.0003591	100	0	0.000816	0	ns	ns
Pedobacter	0.0003357	100	0	0	0.000811	ns	ns

*: Friedman followed by Dunn's multiple comparison test, $p<0.05$. ns: non-significant.

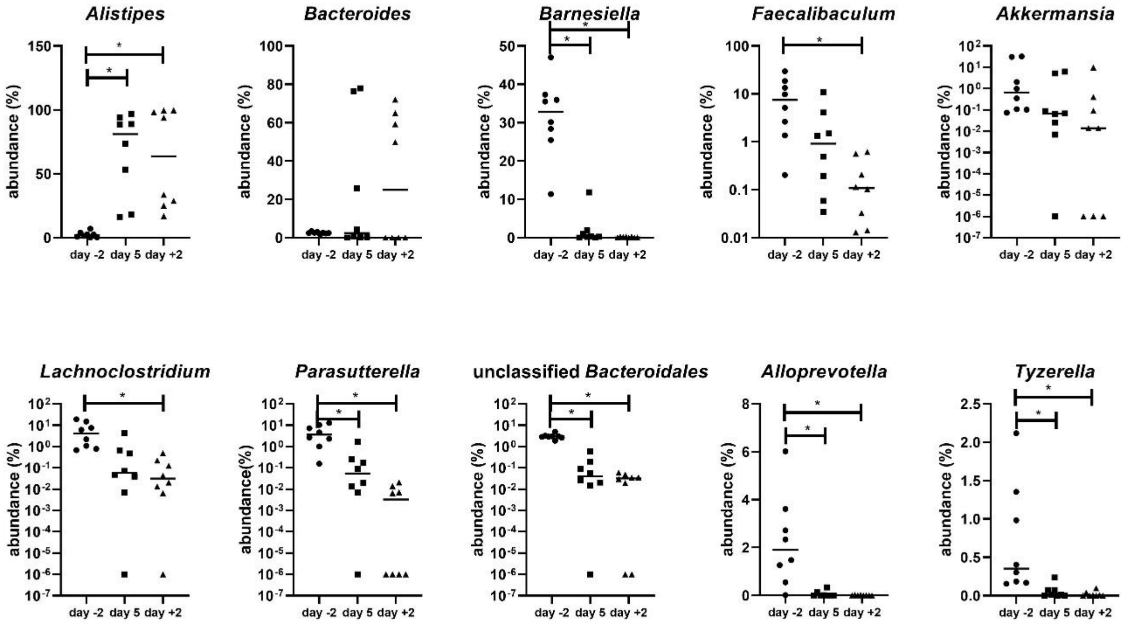

Figure S4: Changes in microbial genera before (day -2), during (day 5) and after (day +2) antibiotic treatment of non-pregnant mice (n=8).

*: Friedman followed by Dunn's multiple comparison's test, $p<0.05$

Figure S4 shows the top 5 bacterial genera of the SIMPER test supplemented with the top 5 bacterial genera of the SIMPER test that were significantly changed at day +2 compared with day -2 for non-pregnant mice. The response of non-pregnant mice was less variable as compared with pregnant mice. All mice increased the genus *Alistipes*, although not all to the same extent. The increase in genus *Alistipes* was either due to an increase in *Alistipes timonensis* or *Alistipes finegoldii*. The two mice with the low increase in genus *Alistipes,* increased genus *Faecalibaculum* (*Faecalibaculum rodentium*). Four mice also increased genus *Bacteroides*. The increase in these specific species was amongst others associated with decreased genera *Barnsesiella*, *Lachnoclostridium*, *Parasutterella*, unclassified *Bacteroidales*, unclassified *Clostridiales* and *Turicibacter*. (See also supplementary table 5).

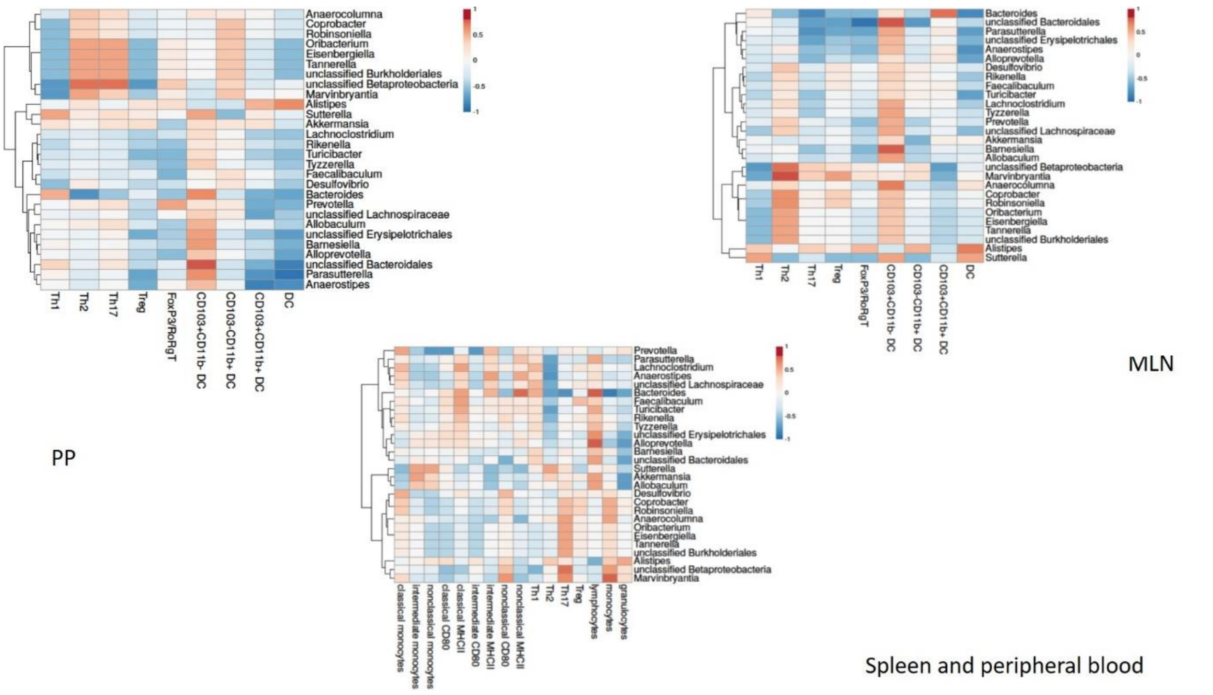

Figure S5: Correlation between immune cell populations in the PP (top left heatmaps), MLN (top right heatmaps) and peripheral cells (lower heatmap) with fecal bacterial genera in non-pregnant mice. The heatmaps show Spearman's correlations coefficients after individual correlation of PPs, MLN or peripheral immune cells (on the x-axis) with bacterial genera that were significantly different between day -2 and day +2 (n=8).

For non-pregnant mice, Pearson's correlation coefficients for PP, MLN and peripheral immune cells with bacterial genera that have changed after antibiotic treatment are shown in heatmaps (figure S5). In the PP, various genera correlated negatively with dendritic cells and CD103+CD11b+ dendritic cells. Various other genera correlated positively with Th2 and Th17 cells. CD103+CD11b- dendritic cells also positively correlated with various bacterial genera. For immune cells in the MLN of non-pregnant mice, various bacterial genera correlated positively with CD103+CD11b- dendritic cells, the most prominent were the genus unclassified *Bacteroidales* and the genus *Barnesiella*. Various genera also correlated positively with Th2 cells. Dendritic cells were negatively correlated with the genera *Alistipes* and *Suttteralla* but negatively correlated to various other genera, such as genera *Bacteroides* and *Parasutterella*. In the spleen, various bacterial genera correlated negatively with Th2 cells, while other correlated positively with Th17 cells. Also granulocytes, monocytes and lymphocytes correlated with various bacterial genera.

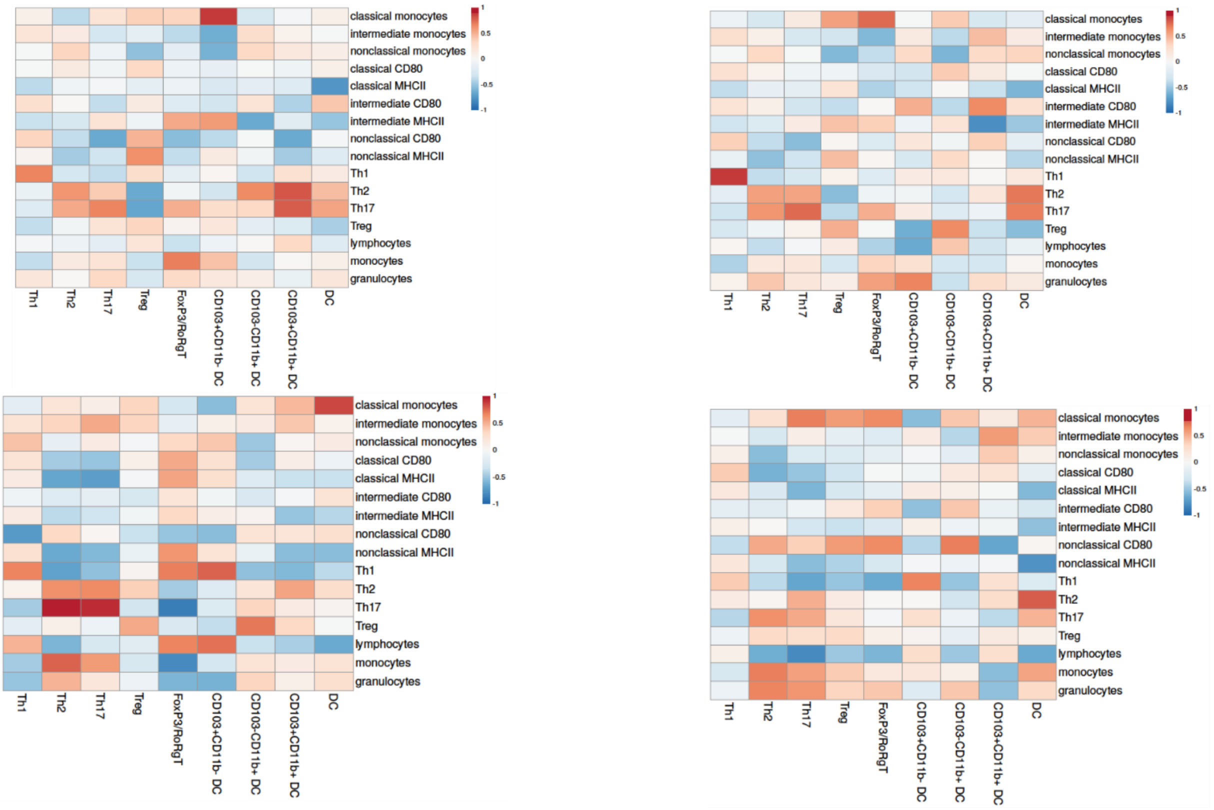

Figure S6: Correlation between immune cell populations in the PPs (left heatmaps) and MLNs (right heatmaps) and peripheral cells in pregnant (top heatmaps) and non-pregnant (lower heatmaps) mice. The heatmaps show Spearman's correlations coefficients after individual correlation of PPs and MLN immune cells (on the x-axis) with peripheral immune cells (on the y-axis).

Intestinal immune responses are under the influence of the microbiome and can regulate peripheral immune responses. Intestinal dendritic cells as well as T cells can circulate to the peripheral circulation, affecting peripheral immune responses. To evaluate the relationship between the intestinal immune cells in the PPs and MLNs with peripheral immune cells, we correlated immune cells from the PPs and MLNs with peripheral immune cells after antibiotic treatment. The heatmaps in figure S6 show the correlation between immune cells in the PPs and MLNs and peripheral immune cells from pregnant mice (top heatmaps). It can be seen that the percentage Th1, Th2 and Th17 cells in the PPs, and even stronger in the MLNs correlated positively with their respective counterparts in the spleen. Various other positive (for instance: CD103+/CD11b+ dendritic cells in the PP correlated positively with Th2 cells and Th17 cells in the spleen) and negative (for instance: dendritic cells in the PP correlated negatively with expression of MHCII on classical monocytes) correlations can be found between PPs and MLNs immune cells and peripheral immune cells.

Also for non-pregnant mice, we found various positive and negative correlations between immune cells in the PPs and MLNs and peripheral immune cells. However, positive

correlations between Th1 cells, Th2 cells and Th17 cells in with their counterparts in the spleen were only found for the PPs and not for the MLNs.
